# *QuickStats*: Age-Adjusted Percentage* of Adults Aged 50–75 Years Who Received the Recommended Colorectal Cancer Screening,^†^ by Sex and Family Income^§^ — National Health Interview Survey, United States, 2021^¶^

**DOI:** 10.15585/mmwr.mm7225a7

**Published:** 2023-06-23

**Figure:**
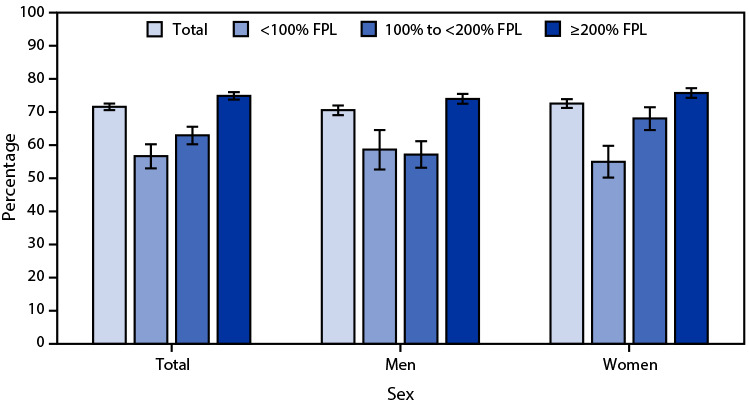
In 2021, 71.6% of adults aged 50–75 years reported they received the recommended colorectal cancer screening, with the percentage increasing with income from 56.7% for those with family incomes <100% of FPL to 63.0% for those with family incomes 100% to <200% of FPL, and 74.9% for those with family incomes ≥200% of FPL. The same pattern by income was found among women, ranging from 55.0% for those with family incomes <100% of FPL to 68.1% for those with family incomes 100% to <200%, and 75.8% for those with family incomes ≥200% of FPL. Among men, the percentage was similar for those with family incomes <100% of FPL (58.7%) and family incomes 100% to <200% of FPL (57.2%), but increased to 74.0% for those with family incomes ≥200% of FPL. Overall, 72.6% of women and 70.6% of men received the recommended screening; the percentage was higher among women than men with family incomes 100% to <200% of FPL (68.1% versus 57.2%), but was similar for the other family income groups.

**Source:** National Center for Health Statistics, National Health Interview Survey, 2021. https://www.cdc.gov/nchs/nhis/index.htm

